# Causal effects and immune cell mediators between prescription analgesic use and risk of infectious diseases: a Mendelian randomization study

**DOI:** 10.3389/fimmu.2023.1319127

**Published:** 2023-12-21

**Authors:** Yi Jin, Xinghao Yu, Jun Li, Mingzhu Su, Xiaomin Li

**Affiliations:** ^1^ The Wujin Clinical College of Xuzhou Medical University, Changzhou, Jiangsu, China; ^2^ Jiangsu Province Key Laboratory of Anesthesiology, Xuzhou Medical University, Xuzhou, Jiangsu, China; ^3^ Department of Pharmacy, Wujin Hospital Affiliated with Jiangsu University, Changzhou, Jiangsu, China; ^4^ National Clinical Research Center for Hematologic Diseases, Jiangsu Institute of Hematology, The First Affiliated Hospital of Soochow University, Suzhou, Jiangsu, China; ^5^ Institute of Blood and Marrow Transplantation, Collaborative Innovation Center of Hematology, Soochow University, Suzhou, Jiangsu, China; ^6^ Center for Genetic Epidemiology and Genomics, School of Public Health, Medical College of Soochow University, Suzhou, Jiangsu, China

**Keywords:** Analgesics, opioids, infection disease, Mendelian randomization, immune cell

## Abstract

**Introduction:**

Clinical observations have found that prolonged use of analgesics increases the incidence of infection. However, the direct causal relationship between prescription analgesic use (PAU) and risk of infection (ROI) remains unclear.

**Methods:**

This study used Mendelian randomization (MR) design to estimate the causal effect of PAU on ROI, as well as their mediating factors. Genetic data on prescription analgesics use and immune cells were obtained from published GWAS. Additionally, data on ROI were extracted from the FinnGen database. Two-sample MR analysis and multivariate MR (MVMR) analysis were performed using inverse variance weighting (IVW) to ascertain the causal association between PAU and ROI. Finally, 731 immune cell phenotypes were analyzed for their mediating role between analgesics and infection.

**Results:**

Using two-sample MR, IVW modeling showed that genetically predicted opioid use was associated with increased risk of pulmonary infection (PI) (OR = 1.13, 95% CI: 1.05–1.21, *p<* 0.001) and upper respiratory infection (URI) (OR = 1.18, 95% CI: 1.08–1.30, *p<* 0.001); non-steroidal anti-inflammatory drugs (NSAIDs) were related to increased risk of skin and subcutaneous tissue infection (OR = 1.21, 95% CI: 1.05–1.39, *p* = 0.007), and antimigraine preparations were linked to a reduced risk of virus hepatitis (OR = 0.79, 95% CI: 0.69–0.91, *p<* 0.001). In MVMR, the association of opioids with URI and PI remained after accounting for cancer conditions. Even with a stricter threshold (*p<* 0.05/30), we found a significant causal association between opioids and respiratory infections (URI/PI). Finally, mediation analyses found that analgesics influence the ROI through different phenotypes of immune cells as mediators.

**Conclusion:**

This MR study provides new genetic evidence for the causal relationship between PAU and ROI, and the mediating role of immune cells was demonstrated.

## 1 Introduction

Since the late 1990s (1), pain has earned the moniker “the fifth vital sign”. Numerous epidemiological studies examining the prevalence of pain have been conducted worldwide. Surveys in various developed countries have reported pain prevalence rates spanning from 23% to 79% (2–8). Consequently, analgesics have become integral to clinical practice. The importance of studying the epidemiology of analgesics is further underscored by the necessity for chronic analgesic utilization in patients with conditions such as cancer pain and chronic pain (9).

Prescription analgesic use (PAU) can have adverse effects on various body systems, including the nervous, digestive, cardiovascular, and endocrine systems (9–11). For instance, opioid medications may lead to sedation and respiratory depression, dependence, addiction, constipation, hormonal imbalances, low blood pressure, and bradycardia (12–16). Additionally, non-steroidal anti-inflammatory drugs (NSAIDs) can contribute to side effects such as gastrointestinal ulcers, fluid retention, and hypertension (17, 18). While there is a known association between PAU and a potential risk of infection (ROI), the nature of this relationship remains unclear. Numerous observational studies have reported a higher incidence of ROI among individuals using PAU (19–22). However, the randomized controlled trials (RCTs) conducted to investigate this relationship had low incidences of infectious complications, resulting in insufficient statistical power to draw definitive conclusions about the link between PAU and ROI (23). Consequently, the connection between PAU and ROI requires further verification. This is particularly critical in cases where the antipyretic effects of non-opioid analgesics and the elevated body temperatures of cancer patients (who are often prescribed opioid analgesics) can obscure infection-related symptoms and potentially increase the risk of mortality. Therefore, clarifying the potential relationship between PAU and ROI is of significant public health importance.

Traditionally, well-designed randomized controlled trials (RCTs) serve as the gold standard for establishing a causal link between PAU and ROI. However, their implementation requires ethical approval, which is not a straightforward process (24). In response to these challenges, Mendelian randomization (MR) analysis has emerged as an advanced epidemiological approach frequently utilized to assess causality between an exposure and an outcome (25). In recent years, advancements in genetic research have shed light on the role of single-nucleotide polymorphisms (SNPs) in mediating responses to analgesic medications and influencing susceptibility to pain (26). These germline genetic variants, inherited randomly from parents to offspring, can serve as instrumental variables (IVs) in MR analyses. These IVs can effectively act as proxies for the exposure of interest, resulting in more robust estimates of causality compared to conventional observational studies (27). There are currently MR studies of opioid analgesics regarding cardiovascular disease and the nervous system (28–31). To date, the association between PAU and ROI has not been investigated in MR studies. This study investigated potential causal associations of PAU [including opioids, NSAIDs, salicylic acid derivatives (SAD), anilides, and antimigraine preparations (AP)] with ROI [including upper respiratory infections (URI), skin and subcutaneous tissue infections (SSTI), intestinal infectious diseases (IID), cystitis, pulmonary infection (PI), and viral hepatitis (VH)] by MR analysis using GWAS summary data from GWAS studies (United Kingdom Biobank and FinnGen database).

## 2 Materials and methods

### 2.1 Study design

We utilized a two-sample MR approach to explore the potential causality of PAU (including opioids, NSAIDs, SAD, and AP) on ROI (including URI, SSTI, IID, VH, PI, and cystitis). We utilized summary data from pre-approved studies, obviating the need for additional ethical clearance for our analysis.

**Figure 1:**
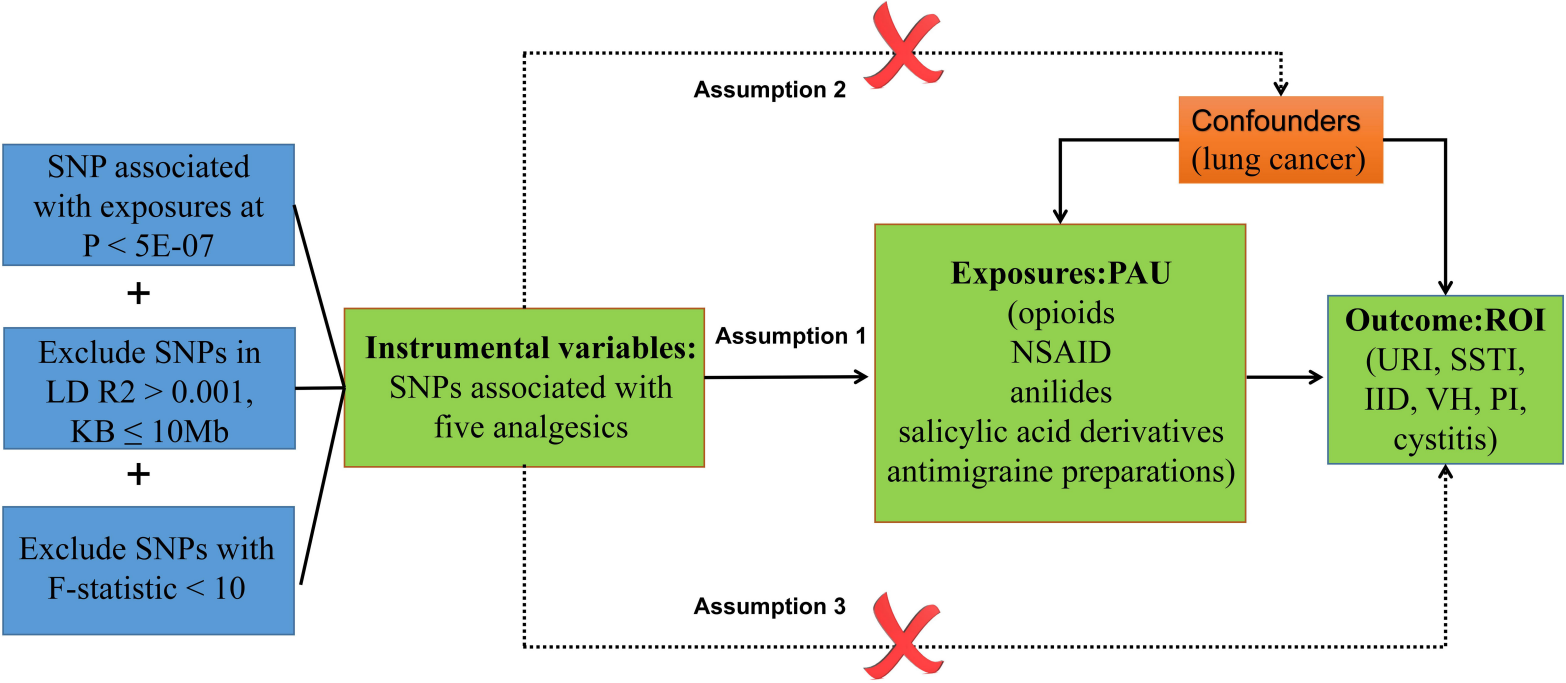
Diagram of the association between PAU and ROI from the two-sample MR study. URI, upper respiratory infections; SSTI, skin and subcutaneous tissue infections; IID, intestinal infectious diseases; VH, viral hepatitis; PI, pulmonary infection.

The MR framework, depicted in **Figure 1**, is grounded in three key assumptions: (1) Relevance Assumption: This initial assumption involves using SNPs significantly associated with exposures as IVs. (2) Independence Assumption: The second assumption stipulates that these SNPs (IVs) should remain uncorrelated with the relevant confounding factors—those factors linked to both the exposure and the corresponding outcome. (3) Exclusivity Assumption: Lastly, the third assumption dictates that the SNPs (IVs) should exclusively influence outcome susceptibility through their direct impact on the exposure, without any other significant associations with the outcome itself (32).

### 2.2 Datasets

**Table 1** provides the data source and definition details. Summary statistics for PAU were derived from a case–control GWAS meta-analysis, encompassing 502,616 participants in the United Kingdom Biobank (UKB) study (26). Of the UKB participants, approximately 54% were female, and the mean age was 56.53 years (with a standard deviation of 8.09). The mean body mass index (BMI) stood at 27.43 (with a standard deviation of 4.80). Notably, the GWAS analysis focused on prescription analgesics, which primarily included opioids, NSAIDs, anilides, SAD, and AP (26).

**Table 1:** Data sources.

Phenotypes	Data source	Phenotypic code	Cases/controls	Ancestry
Exposures				
Opioids	Wu Y. et al. (26)	GCST007936	22,982/55,826	European
NSAIDs	Wu Y. et al. (26)	GCST007934	74,150/90,370	European
SAD	Wu Y. et al. (26)	GCST007937	61,583/50,427	European
Anilides	Wu Y. et al. (26)	GCST007938	83,218/96,592	European
AP	Wu Y. et al. (26)	GCST007939	5,521/114,323	European
Outcome				
URI	FinnGen	J10_UPPERINFEC	69,111/308,166	European
SSTI	FinnGen	L12_INFECT_SKIN	21,308/355,969	European
IID	FinnGen	AB1_INTESTINAL_INFECTIONS	40,881/336,396	European
VH	FinnGen	AB1_VIRAL_HEPATITIS	2,143/375,134	European
PI	FinnGen	PULM_INFECTIONS	117,312/259,965	European
Cystitis	FinnGen	N14_CYSTITIS	18,080/328,001	European
Confounders				
Lung cancer	Sakaue S. et al. (33)	ieu-b-4874	3,791/489,012	European
Mediator				
Immune cells	Orrù V. et al. (34)	GCST0001391 to GCST0002121		European

To minimize sample overlap and to ensure that they were all European samples, summary statistics for ROI were obtained from the FinnGenR9 database (https://r9.finngen.fi/, Public release: 11 May 2023), consisting of up to 377,277 participants (for URI, 69,111 cases and 308,166 control samples; for SSTI, 21,308 cases and 355,969 control samples; for IID, 40,881 cases and 336,396 control samples; for VH, 2,143 cases and 375,134 control samples; for PI, 117,312 cases and 259,965 control samples; for cystitis, 18,080 cases and 328,001 control samples). Based on the cohort information described in the original GWAS analysis, there was no sample overlapping between PAU and ROI in this study.

For the multivariate MR (MVMR) analysis, genetic data for lung cancer were acquired from the MRC-IEU to serve as a confounder (33).

We accessed immunity-wide GWAS data to investigate the mediating role of immune cells. Summary statistics for each immune trait were publicly available through the GWAS Catalog, identified with accession numbers ranging from GCST0001391 to GCST0002121 (34). In total, we incorporated 731 immunophenotypes, which encompassed various categories, including absolute cell counts (*n* = 118), median fluorescence intensities (MFI) reflecting surface antigen levels (*n* = 389), morphological parameters (MP) (*n* = 32), and relative cell counts (*n* = 192). These features, specifically MFI, AC, and RC, comprised a range of immune cells, such as B cells, CDCs, mature T cells, monocytes, myeloid cells, TBNK (T cells, B cells, and natural killer cells), and Treg panels. The MP category included CDC and TBNK panels. To conduct this GWAS analysis, we utilized a dataset consisting of 3,757 Sardinian samples, of which 57% were women. We tested approximately 22 million SNPs genotyped with high-density arrays, while adjusting for sex, age, and age2 (35).

### 2.3 Instrumental variable selection

Owing to the limited number of SNPs reaching genome-wide significance, we employed a more lenient threshold (*p<* 5E-07) and ensured that the selected SNPs were not closely related (at least 10,000 kpb apart) and exhibited a low level of linkage disequilibrium (*R* ^2^ ≤ 0.001). Differently, for reverse MR studies of positive results, when cystitis and VH were used as exposure factors, we set the threshold (*p<* 5E-06) due to the limited number of SNPs satisfying genome-wide significance. To estimate the strength of the selected IVs, variance (*R* ^2^) and *F*-statistics were used (36). *R* ^2^ was calculated as follows: 2×(1−MAF)×MAF×β^2^ (MAF, minor allele frequency; β, effect size on the exposure). The *F*-statistic was computed using the formula *F*=*R* ^2^(*N−K−*1)/[*K*(1*−R* ^2^)]. Here, *R* ^2^ denotes the proportion of exposure variance elucidated by the independent variables, *N* indicates the effective sample size, and *K* represents the quantity of variants contained in the independent variable model. An *F*-statistic > 10 indicates a strong correlation between the IVs and exposure (37). We discarded SNPs with *F*-statistic< 10, suggesting insufficient strength.

### 2.4 MR analysis

MR analysis was performed between PAU and ROI, and for significance results, they were treated alternately as exposure and outcome to disentangle reverse causality (**Figure 1**). In the exposure–outcome analysis, we employed MR with at least two SNPs serving as IVs. Various MR methodologies were applied to infer causal relationships for a total of five PAU and five ROI phenotypes. These methods included inverse variance weighted (IVW), weighted median, MR-Egger, and MR-Pleiotropy RESidual Sum and Outlier (MR-PRESSO). Given that IVW is the most commonly used statistical approach in MR analysis, known for its robust causal assessment even in the absence of directional pleiotropy, it was chosen as the primary method for estimating causal effects (38). Additionally, the weighted median and MR-Egger regression techniques were employed to address potential bias stemming from horizontal pleiotropy and to assess the overall robustness of the IVW method (39). To further examine the exclusivity assumption, we performed the MR-PRESSO test with default parameters to identify outliers possibly influenced by horizontal pleiotropy. We performed power calculations for significant causal relationships (opioid & PI/URI, *p<* 0.05/30) using the online computational tool available at https://shiny.cnsgenomics.com/mRnd/.

### 2.5 Sensitivity analysis

We assessed the heterogeneity of the selected SNPs using Cochrane’s *Q* test (*p<* 0.05). When significant heterogeneity was detected, we applied the random-effects IVW test to ensure more conservative and robust estimates. Additionally, the MR-Egger intercept test was used to evaluate the presence of potential horizontal pleiotropy influencing the MR results. A scatter plot was employed to visualize the causal-effect estimates for individual variants, showing the SNP-outcome associations in relation to SNP-exposure associations. Furthermore, we conducted a “leave-one-out” analysis to examine the stability of the results in the context of a single SNP’s influence and presented the findings in a forest plot.

### 2.6 MVMR analysis

Cancer-related phenotypes were corrected in MVMR analysis. Considering that the majority of patients who clinically use opioids for pain relief have cancer, and recognizing that cancer disrupts local normal tissue defense barriers or obstructs normal tissue and organ lumens, we observed conditions that may facilitate infections. To correct for the causality of opioids with PI and URI, we specifically considered lung cancer as a confounding variable. MVMR analyses utilized the IVW method to assess whether the link between opioid medications and the ROI was primarily influenced by the specific cancer condition at the corresponding site. We incorporate the effects of other traits into linear models according to the following formula and retain the IVs present in the three datasets:


$${\hat{\beta }}^{\text{Infections}}={\hat{\beta }}^{\text{Analgesic}}\theta +{\hat{\beta }}^{\text{Lung cancer}}\mu +\epsilon ,\;\epsilon \sim \;N(0,\;\sigma^{2})$$

where $\hat{\beta }$ is the marginal effect size of instruments, σ2 represents the variance for residual term ϵ , θ represents causal effect of opioids on the infections, and μ represents the causal effect of the lung cancer on infections. MVMR analysis was then performed to estimate the causal effect of opioids on infections after adjusting for confounders.

### 2.7 Mediation analysis

**Figure 2:**
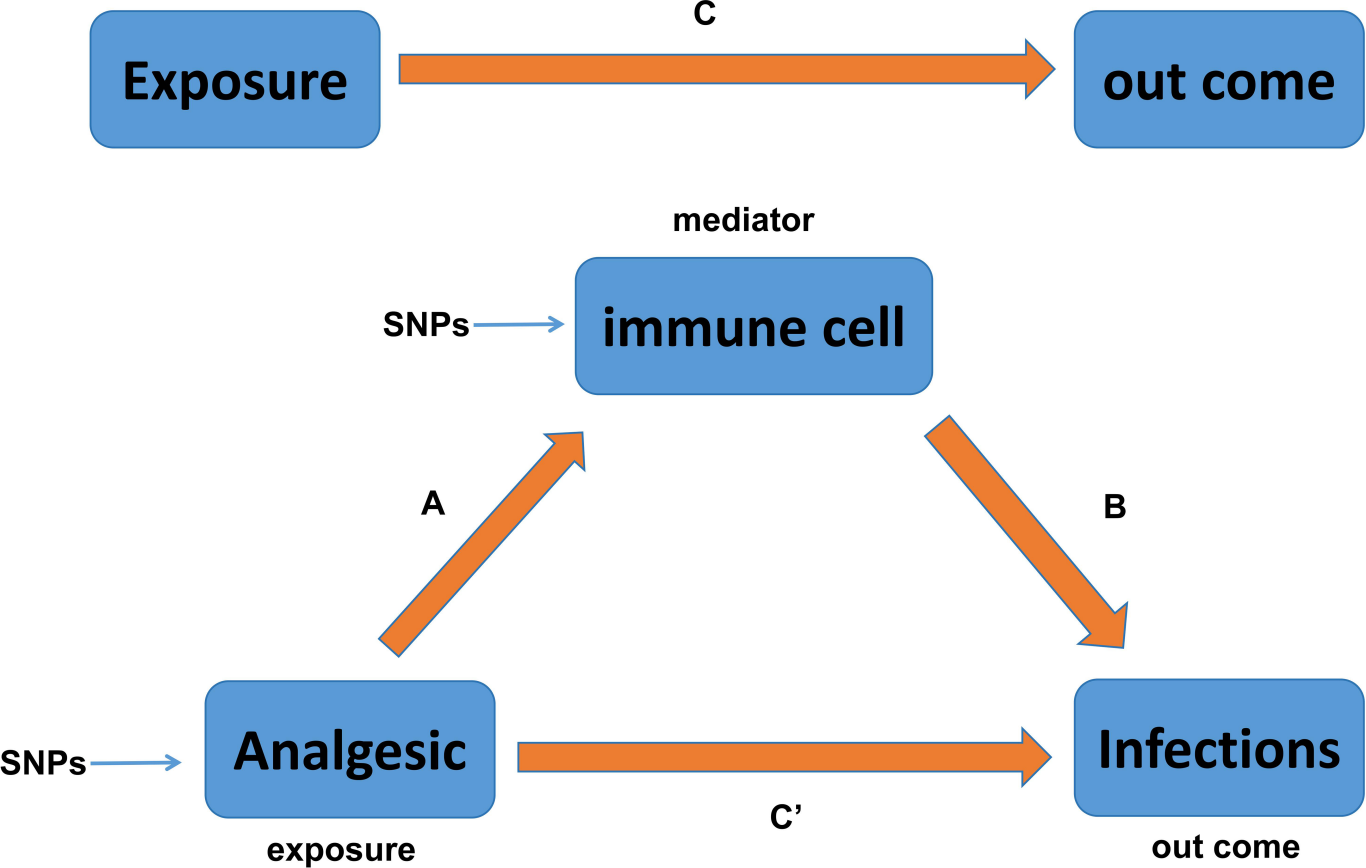
Relationship between analgesic and infections with immune cells as mediators in the Mendelian randomization. Here, C is total effect; C′ is direct effect of analgesic on infections; A is the causal effect of analgesic on immune cells; B is the causal effect of immune cells on infections.

It is well known that the ROI is largely dependent on the body’s immune function, and boosting immune function is often recommended for the clinical management of patients with recurrent infections. Based on the causal relationship between PAU and ROI (opioids and PI/URI; NSAIDs and SSTI; APs and VH) found in the above studies, we next identified two questions that warranted more in-depth investigation: (1) Does PAU (opioids, NSAIDs, and APs) indirectly affect the ROI by modulating immune cells? and (2) What types of immune cell phenotypes might analgesics modulate to indirectly affect the ROI? To investigate these questions, we conducted an immune cell-mediated mediation analysis to estimate the mediating effect of immune cells. This mediation effect estimation and hypothesis testing were carried out within the framework of MR, using summary association statistics of PAU, immune cells, and ROI (**Figure 2**). For detailed methods, we refer to the study by Yu et al. (40). In short, we used the IVW method to estimate the causal effects of opioids and NSAIDs on 731 immune cell phenotypes, and performed MVMR analysis to assess the causal effects of immune cells on URI, PI, SSTI, and VH. The Bootstrap (41, 42) method was finally utilized to test the indirect effect (*H*_0_: *ab* = 0).

All statistical analyses were conducted in R software (V.4.0.4) using the TwoSampleMR package (V.0.5.6) and MR package (V.0.5.1). Given that our MR analysis involved six outcomes, we adjusted the statistical significance level to 8.3E−03 (=0.05/6) to account for multiple hypothesis testing. Additionally, considering that the MR analysis involves 30 sets of analyses, we further adjusted the statistical significance level to 1.7E-03 (=0.05/30) to rigorously control for multiple hypothesis testing. We plan to discuss the results at both significance levels to offer a comprehensive and detailed interpretation of the findings.

## 3 Results

### 3.1 Instrumental variables selection

Based on established quality control criteria, SNPs associated with PAU were selected as IVs (7 SNPs for opioids, 14 SNPs for anilides, 16 SNPs for NSAIDs, 16 SNPs for SAD, and 14 SNPs for AP). In the reverse MR study, SNPs associated with ROI were selected as IVs (12 SNPs for VH, 5 SNPs for SSTI, 14 SNPs for PI, and 12 SNPs for URI). The *F*-statistics of these SNPs exceeded the threshold of 10, signifying their robust representation of PAU in the MR analysis.

### 3.2 Causal estimates of the genetic susceptibility to PAU and ROI

**Figure 3:**
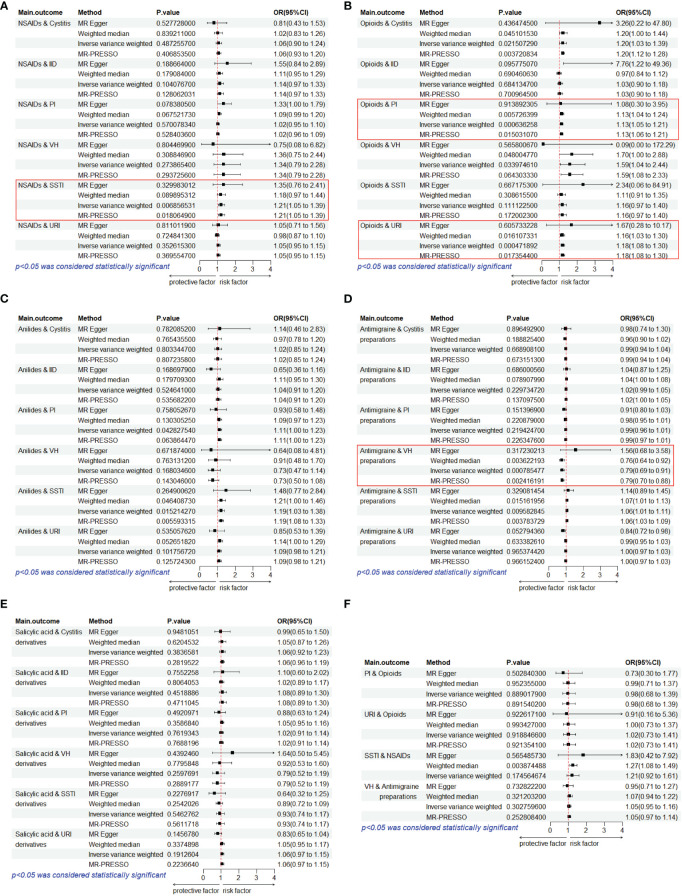
Estimates from Mendelian randomization analysis of PAU and ROI. **(A)** NSAIDs. **(B)** Opioids. **(C)** Anilides. **(D)** Antimigraine preparations. **(E)** Salicylic acid derivatives. **(F)** Reverse MR analysis to rule out reverse causation. OR, odds ratio; CI, confidence interval; URI, upper respiratory infections; SSTI, skin and subcutaneous tissue infections; IID, intestinal infectious diseases; VH, viral hepatitis; PI, pulmonary infection.

We performed MR analysis on five PAU, namely, opioids, anilides, NSAIDs, SAD, and AP, with URI, PI, SSTI, IID, VH, and Cystitis. A total of 30 causal pairs were tested, and 4 of these were statistically significant (*p<* 0.05/6). Finally, four sets of statistically significant samples were used to conduct a reverse MR study, eliminating the impact of reverse causation. In **Figure 3A**, the IVW model indicated that genetically predicted NSAID usage was associated with an increased risk of SSTI (OR = 1.21, 95% CI: 1.05–1.39, *p* = 0.007). As shown in **Figure 3B**, the IVW model revealed that genetically predicted PAU was linked to an increased risk of PI (OR = 1.13, 95% CI: 1.05–1.21, *p<* 0.001) and URI (OR = 1.18, 95% CI: 1.08–1.30, *p<* 0.001). In **Figure 3D**, the IVW model suggested that genetically predicted AP usage was associated with a reduced likelihood of VH (OR = 0.79, 95% CI: 0.69–0.91, *p<* 0.001). Furthermore, MR-Egger regression, Weighted median, and MR-PRESSO analyses consistently supported the directional consistency of the IVW association patterns, confirming the reliable causal relationships between opioids and URI/PI, NSAIDs and SSTI, and AP and VH. Even when employing a more rigorous threshold (*p<* 0.05/30), we consistently detected a notable causal link between opioids and the susceptibility to respiratory system infections (URI/PI). The above results did not reveal reverse causality (*p* > 0.05) in the reverse MR analysis (**Figure 3F**). Our results did not support causal links between other trait pairs (**Figures 3A–F**).

### 3.3 MR sensitivity analysis

In the Cochran’s *Q* test, *p*-values of *Q* statistics were lower than 0.05 in the NSAIDs-IID, Anilides-PI, SAD-IID, SAD-PI, and SAD-SSTI analyses (**Table 2**; **Supplementary Figure S1**). This heterogeneity among IVs justifies the utilization of a random-effects model in these cases. For the remaining IVW analyses, we employed a fixed-effects model. Notably, none of the MR-Egger regression intercepts deviated from 0, indicating an absence of evidence for horizontal pleiotropy in the IVs related to PAU and ROI (all intercept *p* > 0.05) (**Table 2**; **Supplementary Figure S2**). Furthermore, “leave-one-out” analyses confirmed that no specific IV significantly influenced causal inferences (**Supplementary Figure S3**). In summary, the sensitivity analysis reaffirmed the reliability of the MR results.

**Table 2:** Sensitivity analysis for the associations between PAU with ROI.

MR analysis	Heterogeneity test		MR-PRESSO	Pleiotropy test		
	Q_*p*	Q	Global. test. *p*	Egger_ intercept	SE	*p*
NSAIDs-Cystitis	0.798	7.837	0.813	0.013	0.015	0.414
NSAIDs-IID	0.006	29.369	0.009	−0.015	0.014	0.327
NSAIDs-PI	0.687	9.182	0.667	−0.013	0.007	0.090
NSAIDs-VH	0.094	20.069	0.114	0.027	0.051	0.604
NSAIDs-SSTI	0.446	13.026	0.459	−0.005	0.013	0.709
NSAIDs-URI	0.234	16.283	0.248	<0.001	0.009	0.981
Opioids-Cystitis	0.962	1.001	0.960	−0.069	0.093	0.503
Opioids-IID	0.114	8.883	0.149	−0.138	0.064	0.099
Opioids-PI	0.490	4.422	0.552	0.003	0.045	0.948
Opioids-VH	0.547	4.017	0.580	0.196	0.263	0.497
Opioids-SSTI	0.113	0.159	0.159	−0.048	0.125	0.722
Opioids-URI	0.301	6.057	0.356	−0.024	0.063	0.724
Anilides-Cystitis	0.110	19.436	0.133	−0.005	0.021	0.817
Anilides-IID	0.071	21.084	0.058	0.022	0.013	0.124
Anilides-PI	0.018	25.766	0.029	0.008	0.011	0.456
Anilides-VH	0.680	10.167	0.671	0.006	0.046	0.894
Anilides-SSTI	0.904	6.973	0.885	−0.010	0.015	0.526
Anilides-URI	0.117	19.210	0.140	0.011	0.011	0.327
AP-Cystitis	0.468	10.713	0.476	0.001	0.021	0.953
AP-IID	0.862	6.170	0.859	−0.003	0.014	0.842
AP-PI	0.521	10.101	0.521	0.013	0.009	0.205
AP-VH	0.729	6.957	0.762	−0.102	0.062	0.134
AP-SSTI	0.903	5.524	0.901	−0.011	0.019	0.581
AP-URI	0.145	15.909	0.149	0.026	0.012	0.049
SAD-Cystitis	0.815	5.215	0.810	0.005	0.014	0.712
SAD-IID	<0.001	36.040	<0.001	−0.002	0.020	0.931
SAD-PI	0.001	27.323	0.002	0.010	0.011	0.409
SAD-VH	0.354	9.963	0.364	−0.050	0.039	0.236
SAD-SSTI	<0.001	29.241	<0.001	0.026	0.022	0.277
SAD-URI	0.360	9.879	0.390	0.017	0.008	0.059

Q, heterogeneity statistic Q; SE, standard error; OR, odds ratio; CI, confidence interval; URI, upper respiratory infections; SSTI, skin and subcutaneous tissue infections; IID, intestinal infectious diseases; VH, viral hepatitis; PI, pulmonary infection; AP, antimigraine preparations; SAD, salicylic acid derivatives.

### 3.4 Results of the MVMR analysis

**Figure 4:**
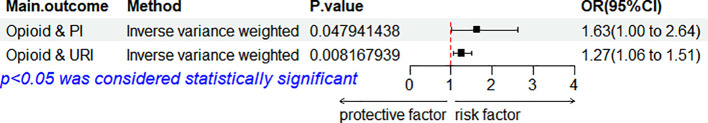
Forest plot for the MVMR considering lung cancer. OR, odds ratio; CI, confidence interval; URI, upper respiratory infections; VH, viral hepatitis; PI, pulmonary infection; MVMR, multivariable MR.

In the MVMR analysis (**Figure 4**), even after adjusting for lung cancer as a confounding factor, a persistent causal effect of genetic susceptibility to opioids on PI was observed (IVW: OR = 1.63, 95% CI: 1.00–2.64, *p* = 0.048), along with a similar effect on URI (IVW: OR = 1.27, 95% CI: 1.06–1.5, *p* = 0.008). Despite the diminished statistical significance following Bonferroni correction, the underlying direction of association remains consistent with our initial findings.

### 3.5 Results of the mediation analysis

**Figure 5:**
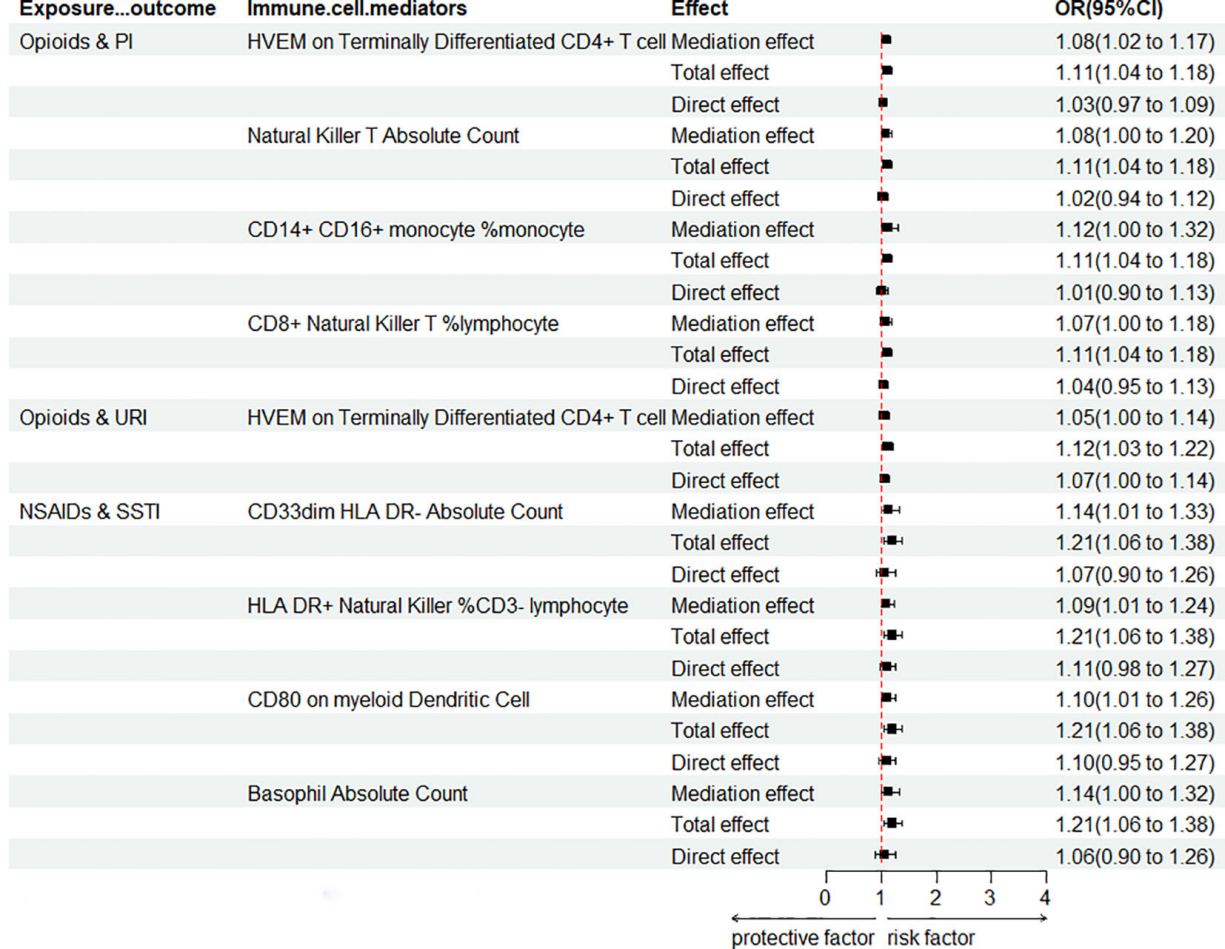
Forest plot for mediation analysis of PAU modulation of immune cells affecting ROI. OR, odds ratio; CI, confidence interval; URI, upper respiratory infections; PI, pulmonary infection; SSTI, skin and subcutaneous tissue infections.

It is widely understood that the immune system plays a crucial role in determining the ROI. To uncover the impact of various analgesic drugs on immune cell phenotypes and their subsequent influence on infection risk, we conducted mediation analyses using 731 immune cell phenotypes with GWAS data as mediators. Given the constraints posed by our sample size and statistical power, we made a deliberate decision not to apply multiple testing adjustments to the corresponding significance levels. Our analysis yielded several noteworthy findings, which have been succinctly summarized (**Figure 5**; **Supplementary Table 10**). The findings indicate that opioids enhance the risk of PI by modulating four immune cell phenotypes (including “HVEM on Terminally Differentiated CD4+ T cell”, “Natural Killer T Absolute Count”, “CD14+ CD16+ monocyte % monocyte”, and “CD8+ Natural Killer T % lymphocyte”), while modulation of “HVEM on Terminally Differentiated CD4+ T cell” immune cells also increases the risk of URI. Moreover, it was concluded that NSAIDs heighten the risk of SSTI by modulating four immune cell phenotypes (including “CD33dim HLA DR- Absolute Count”, “HLA DR+ Natural Killer %CD3- lymphocyte”, “CD80 on myeloid Dendritic Cell”, and “Basophil Absolute Count”). No mediators modulated by APs were found, and it is plausible that its protective effect on VH risk is not mediated by modulation of immune cells.

## 4 Discussion

Analgesics are routinely administered in line with the World Health Organization’s three-step pain relief guidelines (43). Originally, the pain relief ladder consisted of three stages. The first step recommended non-narcotic analgesics for mild pain, while the second and third steps were designed for moderate to severe pain, treatable with strong opioids (44–46). The connection between opioid medications and infection risk has garnered significant attention from the scientific community due to the widespread use of opioids. This study utilized MR methods to investigate the causal relationship between five analgesic drugs and the ROI, encompassing a broader scope beyond opioids alone. To our knowledge, this is the first MR study to evaluate PAU versus ROI. The findings indicated that genetic susceptibility to opioids was linked to an increased risk of PI and URI, while genetic susceptibility to NSAIDs was associated with an elevated risk of SSTI. Conversely, AP was connected to a reduced risk of VH. There is no compelling evidence to support a potential causal link between genetic susceptibility to SAD/anilides and the ROI.

The adverse effects of analgesics are widely recognized. SAD and NSAIDs can cause digestive ulcers complicated by bleeding. Acetaminophen is hepatotoxic, while opioids have potential side effects of addiction, constipation, and neurological disorders (47, 48). These symptoms are entirely predictable and can be prevented with appropriate measures. However, an increasing quantity of studies implies that PAU is strongly associated with enhanced ROI and probably affects immune function (49, 50). Observational studies do not establish causality as RCTs are lacking (23, 51).

In this study, genetically predicted opioids were causally linked to an increased risk of PI and URI. The MVMR analyses found that, removing the interference of lung cancer, opioids have independent causal associations with the risk of PI and URI. In general, this illustrates a significant association between prescription opioid use and an increased risk of respiratory infections. An observational study identified heightened PI incidence associated with opioid use (22), aligning with MR analysis outcomes. Moreover, a study involving patients with rheumatoid arthritis revealed that serious infections, necessitating hospital admission, occurred 40% more frequently during current opioid use compared to non-use periods (52). The use of opioid analgesics in surgical and intensive care settings is associated with a higher risk of secondary infections and mortality (53). Through mediation analyses, we demonstrated that opioids elevate the risk of respiratory infections by influencing immune cells. Additionally, we identified immune cell phenotypes that may be modulated within this pathway, aligning with findings in other studies. Opioid-induced immunosuppression can occur through direct inhibition of immune cells or indirect interactions with the hypothalamic–pituitary–adrenal axis and the sympathetic nervous system (54, 55).

Animal studies have demonstrated that morphine impairs host immune defenses against lung infections through multiple mechanisms, such as impairing the TLR9-NF-KB signaling pathway, which reduces the bacterial clearance of *Streptococcus pneumoniae* from macrophages (56, 57). Morphine (10 nM to 1 μM) disrupted dendritic cells and macrophages producing IL-23, as well as γδ T lymphocytes producing IL-17 in response to lung *S. pneumoniae* infection. This resulted in compromised neutrophil recruitment, leading to a more severe infection lasting up to 24 h post-infection (58). Several studies indicate that opioids may enhance return on investment by impacting the immune system, thereby influencing cancer prognosis (59). The immunosuppressive effect differs depending on the opioid type and is not associated with its potency or duration of action. For instance, hydromorphone, a short-acting and highly potent opioid, does not possess immunosuppressive properties. Conversely, morphine sulfate, which is also a highly potent and short-acting opioid, has been shown to have immunosuppressive effects (60). A retrospective study revealed a higher ROI among cancer patients taking morphine as opposed to those receiving oxycodone, which offers lower immunosuppressive effects (61). Further investigation is necessary in the clinical or laboratory setting to determine the correlation between various opioids medications and the ROI, due to the absence of GWAS data for any individual medication.

The dataset used in this study for NSAID GWAS partially covered anilides, SAD, and AP. Separately, MR analyses were conducted on total NSAIDs, anilides, SAD, and AP. This study offers MR-based evidence of a notable causal link between NSAIDs (IVW: *p* = 0.007) and the risk of SSTI. Additionally, it is worth noting that acetaminophen has been associated with an increased risk of Stevens–Johnson Syndrome (SJS), Toxic Epidermal Necrolysis (TEN), Acute Generalized Exanthematous Pustulosis (AGEP), and Drug Reaction with Eosinophilia and Systemic Symptoms (DRESS) (50, 62, 63). These skin disorders are often caused by drugs, but their pathogenesis is still unclear. In contrast, a comprehensive review of French pharmacovigilance data revealed no apparent relationship between the use of acetaminophen and the risk of SJS/TEN in this extensive national cohort (64). The present study presents further evidence that NSAIDs may increase the risk of SSTI by modulating four phenotypes of immune cells. It has been reported that NSAIDs have varying impacts in adaptive immunity: enhancing cell-mediated immunity but hindering humoral immunity (65). Both French and international research teams, including those from the United States, United Kingdom, and Poland, have conducted studies involving children and adults with bacterial or viral pulmonary infections. These studies have collectively confirmed that exposure to NSAIDs during pulmonary infections increases the risk of severe pulmonary complications (20, 21, 66–71). However, it is essential to note a significant limitation in these studies, which is the potential for protopathic bias. This limitation means that the observed risk could be wrongly attributed to NSAIDs when, in fact, they may merely serve as early markers of the onset of complications (49, 72). MR results also identified AP as a protective factor for VH, which has not been reported and remains to be clinically discovered and validated. Osborne conducted a retrospective study involving 35,370 individuals, comparing those with and without an active aspirin prescription before contracting SARS-CoV-2. The results revealed a notable 32% reduction in the risk of mortality among aspirin users at both 14 and 30 days post-infection (73). This explains the finding that there is no significant causal relationship between SAD and ROI.

Even with a more stringent threshold (*p<* 0.05/30), we still identified a significant causal relationship between opioids and the risk of respiratory system infections (URI/PI). The calculated power values for opioid & PI and opioid & URI were 0.52 and 0.71, respectively, indicating the robustness of the results. Further mediation analysis identified the immune cell phenotype “HVEM on Terminally Differentiated CD4+ T Cell” as a mediator for the association between opioids and PI/URI. Consequently, our study suggests that opioids may increase the risk of respiratory system infections by modulating “HVEM on Terminally Differentiated CD4+ T Cell”. Nevertheless, readers are advised to interpret individual significance values with caution. HVEM (TNFRSF14) belongs to the TNF receptor family and is strongly expressed in immune cells, including resting T cells, B cells, NK cells, Treg cells, monocytes, and dendritic cells. Stromal cells and epithelial cells also express HVEM. In epithelial cells, HVEM plays a crucial role in innate mucosal defense against pathogens. HVEM is involved in diverse immune functions across various cell types. The interaction between HVEM and BTLA was the earliest discovered connection between the Ig superfamily and the TNFR family. HVEM has other ligands besides BTLA, such as CD160, LIGHT, lymphotoxin-alpha (LTα), and herpes simplex virus glycoprotein D. When HVEM binds to LIGHT or LTα, it transmits co-stimulatory signals, while binding to BTLA or CD160 triggers co-inhibitory signals (74). Thus, HVEM is often described as a molecular switch depending on the ligand it engages. A study in cell host and microbe found that HVEM expressed on type 3 innate lymphoid cells (ILC3) is crucial in suppressing pathogenic infections, where LIGHT acts as a ligand for HVEM, activating the HVEM signaling pathway to promote ILC3 secretion of IFN-γ to resist pathogenic infections (75). However, there is currently no research on the interaction between opioid drugs and the LIGHT/HVEM pathway.

This study is the first analysis to use MR to investigate the possible causal connection between genetic susceptibility to various analgesic classes (opioids, anilides, NSAIDs, SAD, and AP) and ROIs (UR, ISSTI, IID, cystitis, PI, and VH). Unlike other observational studies, our MR design sidesteps the usual problems related to confounders and reverse causation. In addition, we utilized extensive GWAS data from the same ethnic group, which yielded strong and dependable IVs and bolstered the causal inference validated through sensitivity analyses. However, MR analyses were not performed for specific medications; for example, opioids, including morphine and oxycodone, were not individually assessed for their association with ROI risk. Lastly, it is worth noting that the GWAS data sample is drawn from European ethnic groups, thus restricting the generalizability of these findings to other ethnic groups.

### 4.1 Limitation

Although this study used genetic data from publicly available GWAS databases, these data may not cover all ethnic groups and populations (only European populations were considered). Therefore, our findings may not be fully generalizable to all populations, especially those underrepresented in databases. Second, our analysis focused primarily on prescription analgesics as an overall category rather than on specific drugs (such as morphine and OxyContin) separately. This may mask differences in infection risk between different analgesics. While MR analysis can provide strong evidence of causality, it relies on several key assumptions, including the absence of genetic confounding factors. If these assumptions are violated, the accuracy of the results may be affected. At the same time, our study assumed a linear relationship between exposure and outcome and could not assess possible nonlinear relationships (e.g., U-shaped). Although our analysis considered 731 immune cell phenotypes as mediating factors, the immune system complexity means that there may be other mediating factors that have not been taken into account. Although our study found an association between analgesic use and infection risk, these results need to be interpreted with caution in a clinical setting, particularly when considering the dose of the drug, frequency of use, and the patient’s specific health status. Finally, this study was designed to generate new hypotheses rather than final definitive conclusions, and without correction for multiple testing, the analysis may be at risk of false positives. Therefore, our results should be considered preliminary and require further validation in future studies.

## 5 Conclusions

This MR study offers fresh genetic evidence supporting the causal link between PAU and ROI. Studies have found a causal relationship between genetic susceptibility to prescription opioid use and the risk of PI and URI, suggesting that opioids increase the risk of respiratory infections by modulating immune cells. NSAIDs have also been found to increase the risk of SSTI by modulating the pathways of immune cells. Conversely, the results showed that AP was a protective factor for VH, which has not been reported. Overall, these results emphasize the importance of improved management and monitoring of analgesic medications by clinicians and pharmacists to reduce the ROI, with a particular focus on patients with pain that is already associated with infection. Further clinical studies and experiments are needed in the future to confirm the findings.

## Data availability statement

The original contributions presented in the study are included in the article/**Supplementary Material**. Further inquiries can be directed to the corresponding author.

## Ethics statement

This study did not require ethical approval from an institutional review board because the summary data were gathered from published studies whose respective institutional review boards had granted approval. The studies were conducted in accordance with the local legislation and institutional requirements. Data were all publicly available, no new data were generated from this study, and ethical approval was provided in all original studies.

## Author contributions

YJ: Conceptualization, Data curation, Formal analysis, Investigation, Methodology, Project administration, Resources, Software, Validation, Visualization, Writing – original draft, Writing – review & editing. XY: Conceptualization, Data curation, Formal analysis, Investigation, Methodology, Project administration, Resources, Software, Validation, Visualization, Writing – original draft, Writing – review & editing. JL: Conceptualization, Data curation, Investigation, Methodology, Project administration, Visualization, Writing – review & editing. MS: Data curation, Methodology, Project administration, Visualization, Writing – review & editing. XL: Conceptualization, Data curation, Formal analysis, Funding acquisition, Investigation, Methodology, Project administration, Resources, Software, Supervision, Validation, Visualization, Writing – original draft, Writing – review & editing.
