# Supplementary material for: Causal effects and immune cell mediators between prescription analgesic use and risk of infectious diseases: a Mendelian randomization study
Source: Front Immunol. 2023 Dec 21;14:1319127. doi: 10.3389/fimmu.2023.1319127 (PMC10772142; doi:10.3389/fimmu.2023.1319127)
Supplement: Supplementary file 1 [file DataSheet_1.doc]

Supplementary Material

***Causal effects and mediating factors between Prescription Analgesic Use and Risk of Infectious Diseases: A*** ***Mendelian Randomization Study***

Yi Jin^1,2†^, Xinhao Yu^3,4,5†^, Jun Li^1,2^, Mingzhu Su^1,2^, Xiaomin Li^1,2*^

**^1^ Department of Pharmacy, Wujin Hospital Affiliated with Jiangsu University,**

**Changzhou 213000, China**

**^2^ The Wujin Clinical College of Xuzhou Medical University**

**^3^ National Clinical Research Center for Hematologic Diseases, Jiangsu Institute of Hematology, The First Affiliated Hospital of Soochow University**

**^4^ Institute of Blood and Marrow Transplantation, Collaborative Innovation Center of Hematology, Soochow University, Suzhou, Jiangsu, China**

**^5^ Center for Genetic Epidemiology and Genomics, School of Public Health, Medical College of Soochow University, Jiangsu 215123, P. R. China**

**† These authors contributed equally to this work and share first authorship.**

* Correspondence:

Xiaomin Li

Email: 404131698@qq.com

**Supplementary figures:**


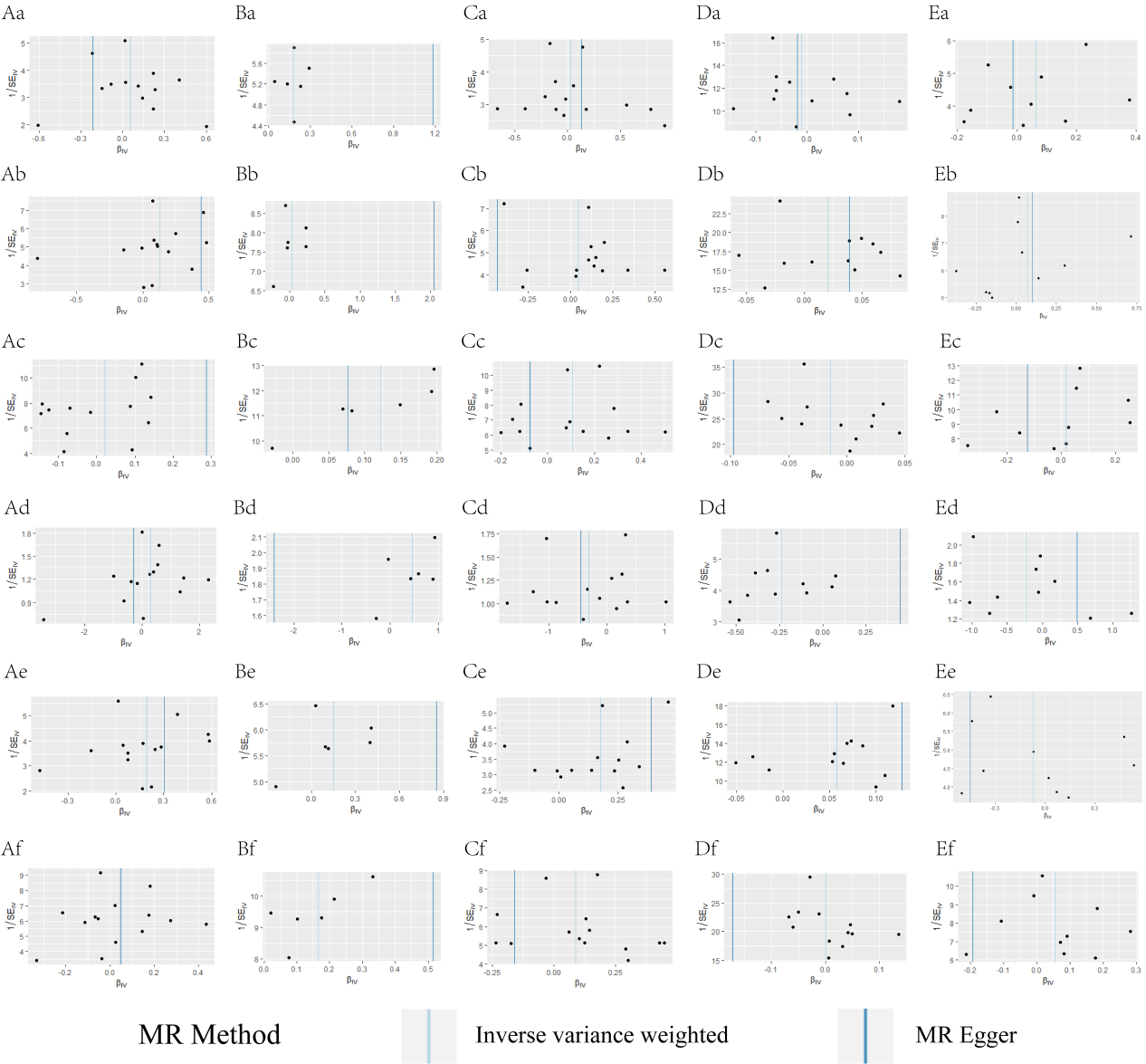


**Supplementary Figure S1.** Funnel plot for IVW and MR-Egger method. (Aa) NSAIDs and cystitis; (Ab) NSAIDs and IID; (Ac) NSAIDs and PI; (Ad) NSAIDs and VH; (Ae) NSAIDs and SSTI; (Af) NSAIDs and URI; (Ba) opioid and cystitis; (Bb) opioid and IID; (Bc) opioid and PI; (Bd) opioid and VH; (Be) opioid and SSTI; (Bf) opioid and URI; (Ca) anilides and cystitis; (Cb) anilides and IID; (Cc) anilides and PI; (Cd) anilides and VH; (Ce) anilides and SSTI; (Cf) anilides and URI; (Da) AP and cystitis; (Db) AP and IID; (Dc) AP and PI; (Dd) AP and VH; (De) AP and SSTI; (Df) AP and URI; (Ea) SAD and cystitis; (Eb) SAD and IID; (Ec) SAD and IID; (Ed) SAD and VH; (Ee) SAD and SSTI; (Ef) SAD and URI.


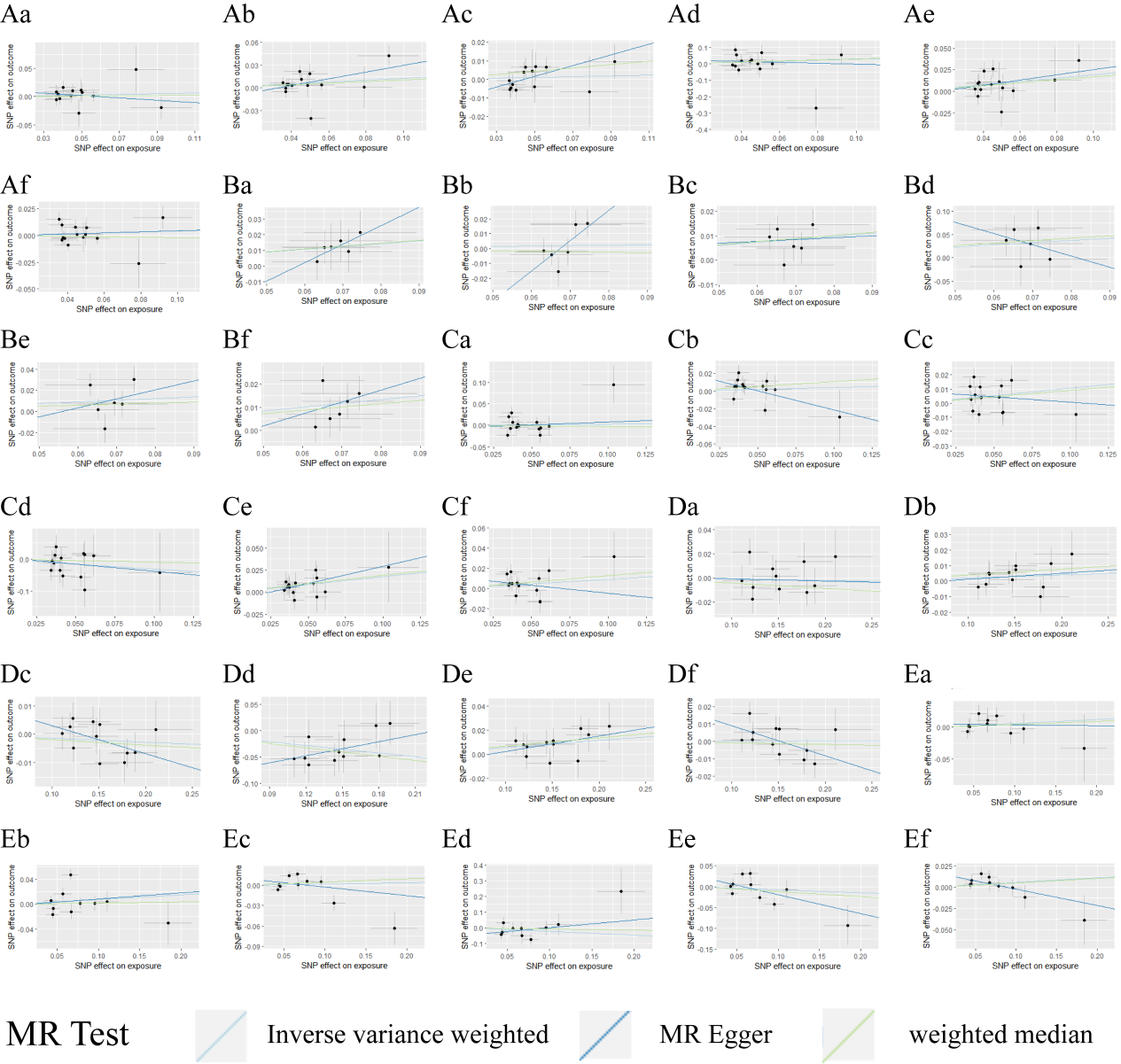


**Supplementary Figure S2.** Scatter plot using all IVs. (Aa) NSAIDs and cystitis; (Ab) NSAIDs and IID; (Ac) NSAIDs and PI; (Ad) NSAIDs and VH; (Ae) NSAIDs and SSTI; (Af) NSAIDs and URI; (Ba) opioid and cystitis; (Bb) opioid and IID; (Bc) opioid and PI; (Bd) opioid and VH; (Be) opioid and SSTI; (Bf) opioid and URI; (Ca) anilides and cystitis; (Cb) anilides and IID; (Cc) anilides and PI; (Cd) anilides and VH; (Ce) anilides and SSTI; (Cf) anilides and URI; (Da)AP and cystitis; (Db) AP and IID; (Dc) AP and PI; (Dd) AP and VH; (De) AP and SSTI; (Df) AP and URI; (Ea) SAD and cystitis; (Eb) SAD and IID; (Ec) SAD and IID; (Ed) SAD and VH; (Ee) SAD and SSTI; (Ef) SAD and URI.


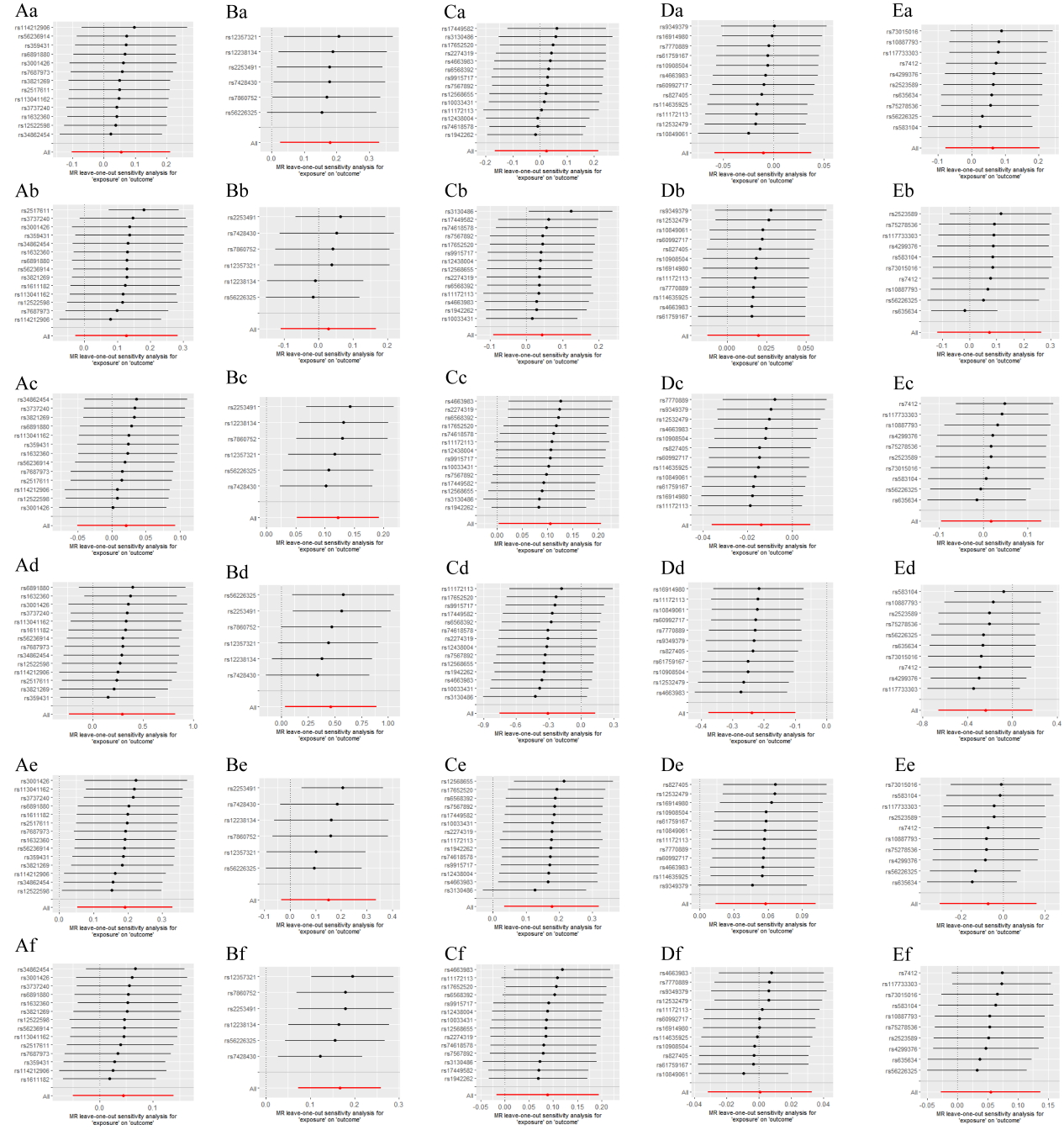


**Supplementary Figure S3.** Leave-one-out sensitivity analysis. (Aa) NSAIDs and cystitis; (Ab) NSAIDs and IID; (Ac) NSAIDs and PI; (Ad) NSAIDs and VH; (Ae) NSAIDs and SSTI; (Af) NSAIDs and URI; (Ba) opioid and cystitis; (Bb) opioid and IID; (Bc) opioid and PI; (Bd) opioid and VH; (Be) opioid and SSTI; (Bf) opioid and URI; (Ca) anilides and cystitis; (Cb) anilides and IID; (Cc) anilides and PI; (Cd) anilides and VH; (Ce) anilides and SSTI; (Cf) anilides and URI; (Da) AP and cystitis; (Db) AP and IID; (Dc) AP and PI; (Dd) AP and VH; (De) AP and SSTI; (Df) AP and URI; (Ea) SAD and cystitis; (Eb) SAD and IID; (Ec) SAD and IID; (Ed) SAD and VH; (Ee) SAD and SSTI; (Ef) SAD and URI.

**Supplementary tables:**

| **Supplementary Table 1: Genetic Variants (n=16) of NSAIDs Used in MR Analyses.** | | | | | | |
| --- | --- | --- | --- | --- | --- | --- |
| **SNPs** | **Effect allele** | **Other allele** | **Beta** | **Se** | **pval** | **F** |
| rs3737240 | T | C | -0.036817 | 0.007049 | 1.80E-07 | 27.3 |
| rs1632360 | G | A | 0.078867 | 0.015176 | 2.00E-07 | 27.0 |
| rs3821269 | A | G | 0.037532 | 0.006923 | 5.90E-08 | 29.4 |
| rs7687973 | C | T | -0.044343 | 0.008324 | 1.00E-07 | 28.4 |
| rs151415 | G | C | 0.042636 | 0.008209 | 2.10E-07 | 27.0 |
| rs56166763 | C | G | -0.040854 | 0.007163 | 1.20E-08 | 32.5 |
| rs12522598 | G | A | 0.045220 | 0.007622 | 3.00E-09 | 35.2 |
| rs6891880 | G | A | -0.038492 | 0.007058 | 4.90E-08 | 29.7 |
| rs359431 | C | T | 0.036749 | 0.006965 | 1.30E-07 | 27.8 |
| rs2517611 | G | A | -0.050484 | 0.008191 | 7.10E-10 | 38.0 |
| rs114212906 | T | C | 0.092068 | 0.016327 | 1.70E-08 | 31.8 |
| rs113041162 | A | G | 0.049852 | 0.009758 | 3.20E-07 | 26.1 |
| rs3001426 | C | T | -0.056192 | 0.007004 | 1.00E-15 | 64.4 |
| rs34862454 | C | T | -0.040197 | 0.007343 | 4.40E-08 | 30.0 |
| rs56236914 | T | C | 0.048602 | 0.008944 | 5.50E-08 | 29.5 |
| rs55938136 | G | A | 0.041652 | 0.008269 | 4.70E-07 | 25.4 |

| **Supplementary Table 2: Genetic variants (n=7) of Opioids used in MR analyses.** | | | | | | |
| --- | --- | --- | --- | --- | --- | --- |
| **SNPs** | **Effect allele** | **Other allele** | **Beta** | **Se** | **pval** | **F** |
| rs2618039 | T | A | 0.061222 | 0.011073 | 3.20E-08 | 30.6 |
| rs7428430 | T | C | -0.065191 | 0.010717 | 1.20E-09 | 37.0 |
| rs2253491 | A | G | -0.066951 | 0.013161 | 3.60E-07 | 25.9 |
| rs56226325 | T | C | -0.074478 | 0.014807 | 4.90E-07 | 25.3 |
| rs7860752 | G | A | 0.069464 | 0.013621 | 3.40E-07 | 26.0 |
| rs12238134 | A | G | -0.071480 | 0.011640 | 8.20E-10 | 37.7 |
| rs12357321 | A | G | 0.063206 | 0.011728 | 7.10E-08 | 29.0 |

| **Supplementary Table 3: Genetic variants (n=14) of Anilides used in MR analyses.** | | | | | | |
| --- | --- | --- | --- | --- | --- | --- |
| **SNPs** | **Effect allele** | **Other allele** | **Beta** | **Se** | **pval** | **F** |
| rs2274319 | T | C | 0.036021 | 0.006854 | 1.50E-07 | 27.6 |
| rs12568655 | A | G | -0.040732 | 0.006694 | 1.20E-09 | 37.0 |
| rs4663983 | G | A | -0.055778 | 0.008286 | 1.70E-11 | 45.3 |
| rs7567892 | T | C | 0.061421 | 0.010950 | 2.00E-08 | 31.5 |
| rs10033431 | C | T | 0.037569 | 0.007417 | 4.10E-07 | 25.7 |
| rs17449582 | T | C | 0.034213 | 0.006762 | 4.20E-07 | 25.6 |
| rs3130486 | T | C | -0.054928 | 0.007387 | 1.00E-13 | 55.3 |
| rs6568392 | G | T | 0.040173 | 0.007890 | 3.60E-07 | 25.9 |
| rs11172113 | C | T | -0.053406 | 0.006642 | 8.90E-16 | 64.7 |
| rs12438004 | C | A | 0.035190 | 0.006670 | 1.30E-07 | 27.8 |
| rs74618578 | G | A | -0.103651 | 0.020559 | 4.60E-07 | 25.4 |
| rs9915717 | A | G | 0.041733 | 0.008158 | 3.10E-07 | 26.2 |
| rs17652520 | A | G | 0.055710 | 0.007781 | 8.10E-13 | 51.3 |
| rs1942262 | A | G | 0.036907 | 0.007204 | 3.00E-07 | 26.2 |

| **Supplementary Table 4: Genetic variants (n=16) of salicylic acid derivatives Used in MR Analyses.** | | | | | | |
| --- | --- | --- | --- | --- | --- | --- |
| **SNPs** | **Effect allele** | **Other allele** | **Beta** | **Se** | **pval** | **F** |
| rs583104 | G | T | -0.078039 | 0.009467 | 1.70E-16 | 67.9 |
| rs10218528 | A | T | -0.041149 | 0.008157 | 4.50E-07 | 25.4 |
| rs4299376 | G | T | 0.045284 | 0.008459 | 8.60E-08 | 28.7 |
| rs2523589 | T | G | 0.044395 | 0.007923 | 2.10E-08 | 31.4 |
| rs117733303 | G | A | 0.184581 | 0.028466 | 8.90E-11 | 42.0 |
| rs74617384 | T | A | 0.105044 | 0.014373 | 2.70E-13 | 53.4 |
| rs56226325 | T | C | -0.056683 | 0.010937 | 2.20E-07 | 26.9 |
| rs75278536 | G | T | -0.067283 | 0.012842 | 1.60E-07 | 27.4 |
| rs28601761 | G | C | -0.060666 | 0.008192 | 1.30E-13 | 54.8 |
| rs1831733 | C | T | 0.045793 | 0.007976 | 9.40E-09 | 33.0 |
| rs635634 | T | C | 0.066638 | 0.010234 | 7.40E-11 | 42.4 |
| rs10887793 | T | G | 0.042721 | 0.008030 | 1.00E-07 | 28.3 |
| rs964184 | G | C | 0.063262 | 0.011595 | 4.90E-08 | 29.8 |
| rs7315004 | A | T | -0.040760 | 0.008085 | 4.60E-07 | 25.4 |
| rs73015016 | A | G | -0.095657 | 0.012271 | 6.40E-15 | 60.8 |
| rs7412 | T | C | -0.110777 | 0.014703 | 4.90E-14 | 56.8 |

| **Supplementary Table 5: Genetic variants (n=14) of antimigraine preparations used in MR analyses.** | | | | | | |
| --- | --- | --- | --- | --- | --- | --- |
| **SNPs** | **Effect allele** | **Other allele** | **Beta** | **Se** | **pval** | **F** |
| rs61759167 | T | C | 0.151167 | 0.023280 | 8.40E-11 | 42.2 |
| rs10908504 | C | A | 0.122428 | 0.020323 | 1.70E-09 | 36.3 |
| rs4663983 | G | A | -0.188979 | 0.024390 | 9.30E-15 | 60.0 |
| rs60992717 | A | G | 0.110503 | 0.020504 | 7.10E-08 | 29.0 |
| rs114635925 | A | G | 0.210893 | 0.040398 | 1.80E-07 | 27.3 |
| rs9349379 | G | A | -0.180234 | 0.019655 | 4.70E-20 | 84.1 |
| rs7770889 | T | C | 0.150991 | 0.020326 | 1.10E-13 | 55.2 |
| rs74809038 | G | A | 0.158410 | 0.026817 | 3.50E-09 | 34.9 |
| rs12532479 | C | T | 0.177533 | 0.031446 | 1.60E-08 | 31.9 |
| rs16914980 | C | T | -0.122270 | 0.022149 | 3.40E-08 | 30.5 |
| rs7852872 | G | C | -0.142738 | 0.020058 | 1.10E-12 | 50.6 |
| rs827405 | T | C | 0.147334 | 0.024346 | 1.40E-09 | 36.6 |
| rs10849061 | C | T | 0.119227 | 0.019418 | 8.30E-10 | 37.7 |
| rs11172113 | C | T | -0.143504 | 0.019624 | 2.60E-13 | 53.5 |

| **Supplementary Table 6: Genetic variants (n=12) of VH used in MR analyses.** | | | | | | |
| --- | --- | --- | --- | --- | --- | --- |
| **SNPs** | **Effect allele** | **Other allele** | **Beta** | **Se** | **pval** | **F** |
| rs35287180 | A | G | -0.337935 | 0.073237 | 0.000004 | 21.3 |
| rs140095464 | A | G | 1.289170 | 0.276750 | 0.000003 | 21.7 |
| rs62356114 | T | C | 0.344164 | 0.074663 | 0.000004 | 21.2 |
| rs67914330 | C | T | 0.294611 | 0.063939 | 0.000004 | 21.2 |
| rs9378487 | C | T | -0.418539 | 0.087947 | 0.000002 | 22.6 |
| rs118022964 | A | T | 0.274742 | 0.058375 | 0.000003 | 22.2 |
| rs7006620 | T | C | -0.260286 | 0.056707 | 0.000004 | 21.1 |
| rs112398420 | T | C | 0.237780 | 0.051412 | 0.000004 | 21.4 |
| rs74659626 | A | G | -0.192951 | 0.039592 | 0.000001 | 23.8 |
| rs3744588 | C | T | -0.150554 | 0.031325 | 0.000002 | 23.1 |
| rs117209342 | C | G | -1.441090 | 0.308635 | 0.000003 | 21.8 |
| rs77013778 | T | C | -0.561614 | 0.116917 | 0.000002 | 23.1 |

| **Supplementary Table 7: Genetic variants (n=12) of URI used in MR analyses.** | | | | | | |
| --- | --- | --- | --- | --- | --- | --- |
| **SNPs** | **Effect allele** | **Other allele** | **Beta** | **Se** | **pval** | **F** |
| rs7527638 | T | C | -0.0350394 | 0.006203 | 1.62E-08 | 31.9 |
| rs150649461 | C | G | 0.0937161 | 0.0183938 | 3.49E-07 | 26.0 |
| rs2139846 | G | A | 0.0679664 | 0.0123687 | 3.91E-08 | 30.2 |
| rs1041973 | A | C | -0.0468047 | 0.00760226 | 7.43E-10 | 37.9 |
| rs1398948 | C | T | 0.0335251 | 0.00665077 | 4.64E-07 | 25.4 |
| rs2735076 | G | A | 0.0463637 | 0.00840661 | 3.48E-08 | 30.4 |
| rs586610 | C | T | 0.0654949 | 0.00745587 | 1.57E-18 | 77.2 |
| rs7454285 | T | C | -0.0440771 | 0.00870069 | 4.06E-07 | 25.7 |
| rs2331208 | C | T | -0.0345202 | 0.00664044 | 2.01E-07 | 27.0 |
| rs7848215 | T | C | 0.0389915 | 0.0071319 | 4.57E-08 | 29.9 |
| rs11116537 | T | C | 0.0332336 | 0.00642666 | 2.33E-07 | 26.7 |
| rs3184504 | C | T | -0.0400967 | 0.00619895 | 9.91E-11 | 41.8 |

| **Supplementary Table 8: Genetic variants (n=5) of SSTI used in MR analyses.** | | | | | | |
| --- | --- | --- | --- | --- | --- | --- |
| **SNPs** | **Effect allele** | **Other allele** | **Beta** | **Se** | **pval** | **F** |
| rs41315573 | T | G | -0.112792 | 0.0222801 | 4.14E-07 | 25.6 |
| rs2760985 | A | G | 0.102648 | 0.0136893 | 6.46E-14 | 56.2 |
| rs9690826 | G | A | 0.0766478 | 0.0135914 | 1.71E-08 | 31.8 |
| rs73169662 | C | T | -0.0759233 | 0.014831 | 3.07E-07 | 26.2 |
| rs7146787 | G | A | 0.059561 | 0.0117906 | 4.38E-07 | 25.5 |

| **Supplementary Table 9: Genetic variants (n=14) of PI used in MR analyses.** | | | | | | |
| --- | --- | --- | --- | --- | --- | --- |
| **SNPs** | **Effect allele** | **Other allele** | **Beta** | **Se** | **pval** | **F** |
| rs12741825 | T | C | 0.0357166 | 0.00528374 | 1.38E-11 | 45.7 |
| rs72820194 | T | C | -0.0363875 | 0.00703706 | 2.33E-07 | 26.7 |
| rs4848344 | G | T | 0.027669 | 0.00526399 | 1.47E-07 | 27.6 |
| rs144537600 | C | T | 0.0773203 | 0.0152224 | 3.79E-07 | 25.8 |
| rs586610 | C | T | 0.0513079 | 0.00619404 | 1.20E-16 | 68.6 |
| rs4720143 | G | T | -0.027113 | 0.00508047 | 9.46E-08 | 28.5 |
| rs118105263 | T | C | -0.0912363 | 0.0167458 | 5.09E-08 | 29.7 |
| rs112894662 | G | A | -0.0968832 | 0.0169414 | 1.07E-08 | 32.7 |
| rs1904721 | G | A | -0.029342 | 0.00564888 | 2.05E-07 | 27.0 |
| rs7074653 | C | G | -0.0587951 | 0.0103766 | 1.46E-08 | 32.1 |
| rs28490880 | C | A | -0.0270851 | 0.00532949 | 3.73E-07 | 25.8 |
| rs3184504 | C | T | -0.0301108 | 0.00512717 | 4.29E-09 | 34.5 |
| rs7359623 | T | C | -0.0261484 | 0.00507714 | 2.60E-07 | 26.5 |
| rs17410557 | C | T | 0.0262095 | 0.00516365 | 3.86E-07 | 25.8 |

| **Supplementary Table 10: Mediating role of immune cells between PAUs and ROIs** | | | | | | | | |
| --- | --- | --- | --- | --- | --- | --- | --- | --- |
| Exposure | Outcome | Mediator  (immune cell) | Mediation effect  OR (95% CI) | Total effect  OR (95% CI) | Direct effect  OR (95% CI) | indirect.product.1 | indirect.product.2 | Proportion (%) |
| Opioids | PI | HVEM on Terminally Differentiated CD4+ T cell | 1.078 (1.017 ~ 1.166) | 1.111 (1.042 ~ 1.185) | 1.031 (0.972 ~ 1.094) | -0.146 | -0.513 | 71.1% |
| Opioids | PI | Natural Killer T Absolute Count | 1.084 (1.005 ~ 1.198) | 1.111 (1.042 ~ 1.185) | 1.025 (0.936 ~ 1.123) | -0.374 | -0.215 | 76.5% |
| Opioids | PI | CD14+ CD16+ monocyte %monocyte | 1.120 (1.003 ~ 1.319) | 1.111 (1.042 ~ 1.185) | 1.009 (0.902 ~ 1.129) | -0.345 | -0.329 | 107.7% |
| Opioids | PI | CD8+ Natural Killer T %lymphocyte | 1.072 (1.001 ~ 1.181) | 1.111 (1.042 ~ 1.185) | 1.036 (0.952 ~ 1.127) | -0.234 | -0.299 | 66.5% |
| Opioids | URI | HVEM on Terminally Differentiated CD4+ T cell | 1.051 (1.002 ~ 1.135) | 1.122 (1.030 ~ 1.223) | 1.068 (1.004 ~ 1.135) | -0.135 | -0.372 | 43.4% |
| NSAIDs | SSTI | CD33dim HLA DR- Absolute Count | 1.135 (1.010 ~ 1.331) | 1.211 (1.058 ~ 1.385) | 1.067 (0.905 ~ 1.258) | 0.172 | 0.736 | 66.3% |
| NSAIDs | SSTI | HLA DR+ Natural Killer %CD3- lymphocyte | 1.089 (1.011 ~ 1.239) | 1.211 (1.058 ~ 1.385) | 1.112 (0.977 ~ 1.267) | -0.188 | -0.452 | 44.4% |
| NSAIDs | SSTI | CD80 on myeloid Dendritic Cell | 1.102 (1.009 ~ 1.262) | 1.211 (1.058 ~ 1.385) | 1.099 (0.952 ~ 1.269) | 0.210 | 0.463 | 50.7% |
| NSAIDs | SSTI | Basophil Absolute Count | 1.139 (1.002 ~ 1.325) | 1.211 (1.058 ~ 1.385) | 1.063 (0.895 ~ 1.262) | 0.176 | 0.741 | 68.2% |
